# Supplementary material for: A cascade training protocol for schoolteachers on oral health: Investigating the influence of primary and secondary mentors
Source: PLoS One. 2024 Aug 29;19(8):e0309671. doi: 10.1371/journal.pone.0309671 (PMC11361646; doi:10.1371/journal.pone.0309671)
Supplement: Attachment — Submitted filename: Response to Reviewers.pdf [file pone.0309671.s002.pdf]

Centre for Dental Education and Research

All India Institute of Medical Sciences New Delhi

Dear Dr. Apurva Pandya,

Thank you for your valuable feedback on our manuscript. We have addressed the points raised in your review as follows:

Suggestion 1: Formatting and Style Requirements

Action Taken: We have ensured that our manuscript meets the PLOS ONE style requirements, including those for file naming. The revised manuscript adheres to the guidelines provided in the PLOS ONE style templates.

Suggestion 2: Data Availability

Action Taken: In accordance with PLOS ONE's data availability policy, we confirm that de-identified participant data will be made available on reasonable request from the corresponding author. The data underlying our findings will be deposited in a public data repository and made accessible in a masked data sheet to ensure participant confidentiality.

Suggestion 3: Reference List Review

Action Taken: We have reviewed our reference list thoroughly to ensure it is complete and correct. We did not find any retracted papers among our citations. Therefore, no retractions are cited in our manuscript, and all references are up-to-date and relevant.

Thank you for your consideration. We look forward to your positive response.

Kind regards,

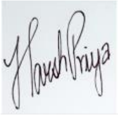

Dr. Harsh Priya,
Additional Professor,
Department of Public Health Dentistry
Centre for Dental Education and Research,
All India Institute of Medical Sciences, New Delhi.
