# Supplementary material for: A cascade training protocol for schoolteachers on oral health: Investigating the influence of primary and secondary mentors
Source: PLoS One. 2024 Aug 29;19(8):e0309671. doi: 10.1371/journal.pone.0309671 (PMC11361646; doi:10.1371/journal.pone.0309671)
Supplement: Attachment — Submitted filename: Response to reviewers.docx [file pone.0309671.s001.docx]

**Response to reviewers**

1.Comment from the editor: **The ethics approval document of the earlier submission (PONE-D-24-15637) is dated 26 May 2023, while the completed collecting patient samples in January 2023.**

**Response**: Contrary to the dates indicated in our initial submission, the patient recruitment process commenced subsequent to obtaining approval from our local ethics committee. Regrettably, there was an inadvertent typographical error in our submission that misrepresented the sequence of events.

To offer clarity, the initial patient recruitment occurred in the form of requesting teachers to complete a pre-training questionnaire on 1/11/23, followed by a training session conducted on 6/11/23. These activities transpired after the receipt of ethics approval on 26 May 2023. Subsequently, two teachers from this training further disseminated the training to their monitors and class students, with data collection commencing in January 2024 which we used for pilot analysis.

In an effort to rectify the misunderstanding and provide evidence of our adherence to ethical guidelines, we have included the following supporting documents:

1.Permission from our headquarters, dated 4/8/23 demonstrating that the recruitment process occurred subsequent to the receipt of ethics approval. (Attached below)

2.A masked data collection sheet with timestamps indicating data collection prior to the first training session available in the following link:

<https://docs.google.com/spreadsheets/d/1_2l68jixqV2RXFAuRPUfiaLMTsx18heV/edit?usp=sharing&ouid=115714412373196002040&rtpof=true&sd=true>

3.Screenshots of WhatsApp messages with timestamps, announcing the teachers to fill the pre-training questionnaire and detailing the date of the first training session. These timestamps unequivocally confirm that all activities occurred following the receipt of ethics approval.
